# Loss of the novel mitochondrial protein FAM210B promotes metastasis via PDK4-dependent metabolic reprogramming

**DOI:** 10.1038/cddis.2017.273

**Published:** 2017-06-08

**Authors:** Shujuan Sun, Jia Liu, Meisong Zhao, Yingyan Han, Pingbo Chen, Qingqing Mo, Beibei Wang, Gang Chen, Yong Fang, Yuan Tian, Jianfeng Zhou, Ding Ma, Qinglei Gao, Peng Wu

**Affiliations:** 1 The Key Laboratory of Cancer Invasion and Metastasis of the Ministry of Education of China, Tongji Hospital, Tongji Medical College, Huazhong University of Science and Technology, Wuhan, Hubei 430030, P.R. China; 2 Department of hematology, Tongji Hospital, Tongji Medical College, Huazhong University of Science and Technology, Wuhan, Hubei, P.R. China

## Abstract

Recent advances in tumor metabolism have revealed that metabolic reprogramming could dramatically promote caner metastasis. However, the relation and mechanism between metastasis and metabolic reprogramming are not thoroughly explored. Cell proliferation, colony formation, and invasion analysis were performed to evaluate the role of FAM210B in human cancer cells. Human ovarian cancer xenograft model was used to determine the effects of inhibiting FAM210B by shRNA on tumor metastasis. Microarray analysis was used to determine the target genes of FAM210B. FAM210B cellular localization was performed by mitochondria isolation and mitochondria protein extraction. To detect FAM210B-mediated metabolic reprogramming, oxygen consumption rate and extracellular acidification rate were measured. Our previous study screened a novel cancer progression-suppressor gene, FAM210B, which encodes an outer mitochondrial membrane protein, by the suppression of mortality by antisense rescue technique (SMART). Here we demonstrated that FAM210B loss was significantly associated with cancer metastasis and decreased survival in a clinical setting. Additionally, it was found that low expression of FAM210B was significantly correlated with decreased survival and enhanced metastasis *in vivo* and *in vitro*, and the loss of FAM210B led to an increased mitochondrial respiratory capacity and reduced glycolysis through the downregulation of pyruvate dehydrogenase kinase 4 (PDK4), which activated the EMT program and enhanced migratory and invasive properties. Collectively, our data unveil a potential metabolic target and mechanism of cancer metastasis.

Ovarian cancer is the most lethal gynecological malignancy.^1^ Although ongoing efforts have improved the early diagnosis, metastases decrease the cure rate substantially.^2, 3^

An emerging hallmark of cancer is the reprogramming of energy metabolism to fuel tumor cell growth, division, and survival.^4, 5, 6^ Recent studies have revealed that enhanced mitochondrial function have important roles in tumor proliferation and metastasis.^7, 8, 9, 10^ The metabolic status malignant tumors exploit to regulate growth and proliferation is becoming increasingly clear.^11^ However, how cancer cells alter the metabolic program and affect metastasis is not well understood.

Our previous study screened the novel gene FAM210B from the library involved in the tumor-selective killing of trichostatin A (TSA) by suppression of mortality by antisense rescue technique (SMART).^12^ In this study, further analysis indicated that FAM210B was a novel mitochondria outer membrane protein and low expression of FAM210B obviously promoted malignant metastasis *in vitro* and *in vivo.* Depletion of FAM210B in ovarian cancer cells displayed elevated mitochondrial respiration and decreased glycolysis relative to control cells via the suppression of its downstream target, pyruvate dehydrogenase kinase 4 (PDK4). Thus our results suggested that low expression of FAM210B promoted metastasis through metabolic reprogramming.

## Results

### Low expression of FAM210B was associated with metastasis and decreased survival

**Figure 1:**
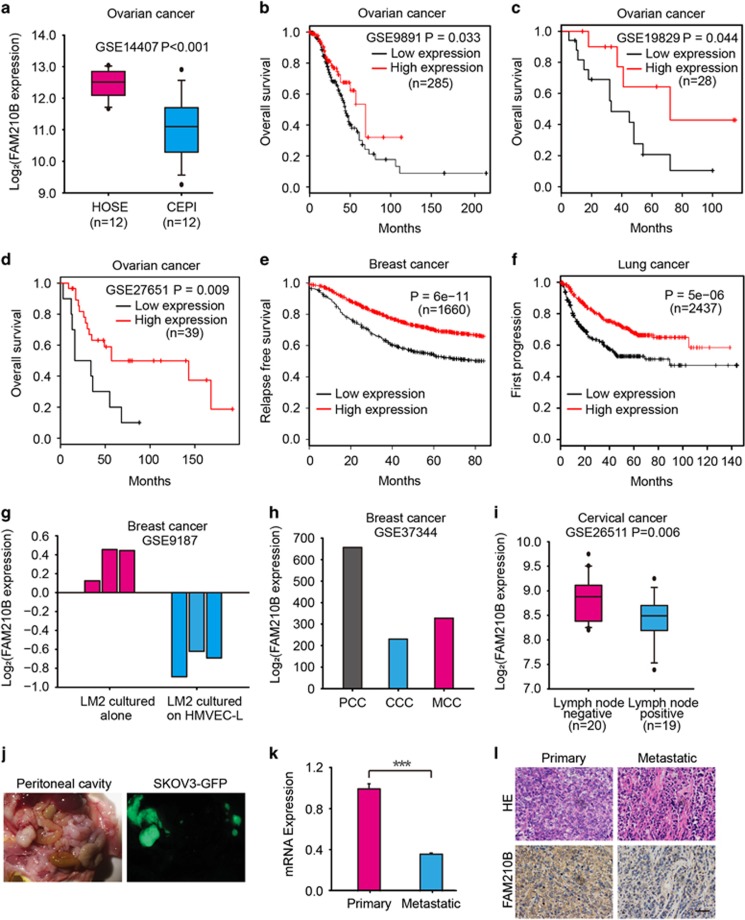
Low expression of FAM210B is associated with poor prognosis in cancer patients. (**a**) FAM210B expression levels in ovarian cancer epithelia (CEPI) samples and normal human ovarian surface epithelia (HOSE) (GSE 14407data set). The expression levels are presented as boxplots and were compared using unpaired Student’s *t*-test. KM analysis of (**b**) overall survival in the GSE9891 data set, (**c**) in the GSE19829 data set, and (**d**) in the GSE27651 data set. (**e**) Recurrence-free survival in all breast cancer data sets and (**f**) first progression survival in all lung adenocarcinoma data sets in KM plotter based on FAM210B expression in the primary tumor patients. (**g**) FAM210B expression in the breast cancer cell line LM2 cultured alone or with HMVEC-L (human lung microvascular endothelial cells) (GSE9187 data set). (**h**) FAM210B expression in circulating cancer cells (CCC), cancer cells from primary tumors (PCC), and metastatic lung cells (MCC) in a 4T1 mammary epithelial cancer cell orthotopic mouse model (GSE37344 data set). (**i**) FAM210B expression in cervical cancer patients with negative and positive lymph nodes (GSE26511 data set). The expression levels are presented as boxplots and were compared using unpaired Student’s *t*-test. (**j**) Representative image of SKOV3-luc-GFP primary tumor and peritoneal cavity metastasis. (**k**) mRNA expression and (**l**) immunohistocytochemistry (IHC) scores of FAM210B in primary tumor and metastatic site

**Figure 2:**
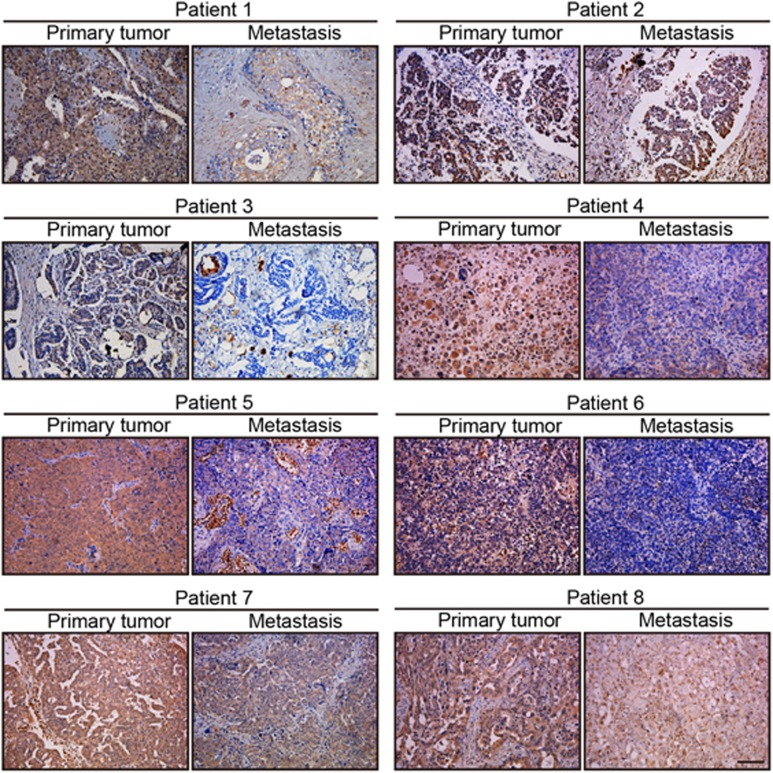
FAM210B expression is reduced in the metastatic site of ovarian cancer. Representative images of FAM210B staining in primary human ovarian tumors and the metastatic site are shown. The scale bar represents 50 mm and applies to all panels

We previously screened a new gene, FAM210B, from the library involved in the tumor-selective killing of trichostatin A (TSA) by SMART.^12^ In this study, we also found that the expression of FAM210B was selectively upregulated by TSA in tumor cells (Supplemenetary Figures S1A and B). As in our previous study, the role of FAM210B in TSA-induced apoptosis was tested. Cells were stably infected with lentivirus expressing either a small hairpin RNA (shRNA) non-targeting control or two specific shRNAs that knock down FAM210B (Supplemenetary Figure S1C). TSA resulted in profoundly reduced proliferation and colony formation in shScramble cells but not in shFAM210B cells (Supplemenetary Figures S1D and E). Decreased expression of FAM210B also moderately inhibited TSA-induced apoptosis, suggesting that FAM210B was essential for apoptosis mediated by TSA (Supplemenetary Figure S1F). On the other hand, we also found that low expression of FAM210B was significantly associated with metastasis and decreased survival (Figure 1). Because FAM210B can be regulated at the mRNA level (Supplemenetary Figure S1A), the available public gene expression databases were surveyed. In the ovarian cancer data set GSE14407, FAM210B expression was significantly reduced in ovarian cancer epithelia (CEPI) samples compared with normal human ovarian surface epithelia (HOSE) (Figure 1a). Furthermore, TCGA data showed that FAM210B mRNA tended to be lower in many types of cancer than in normal cells (Supplemenetary Figure S2). Based on the above results, we further elucidated the clinical outcome relevance of FAM210B in various cancer patients using Kaplan–Meier (KM) plots (http://www.kmplot.com). In the ovarian cancer data sets GSE9891, GSE19829 and GSE27651, analysis showed that low expression of FAM210B was significantly correlated with decreased overall survival (Figures 1b–d). All of the breast cancer and lung adenocarcinoma data sets in KM plotter also revealed that patients with low FAM210B had a significantly poor prognosis (Figures 1e and f). Thus it supposed that FAM210B may have a crucial role in cancer progress and metastasis. Consistent with the above results, in the breast cancer metastatic model of GSE9187 and GSE37344, FAM210B expression was significantly low in the metastatic phenotype (Figures 1g and h). GSE26511 revealed that cervical cancer patients with positive lymph nodes had a significantly lower FAM210B expression (Figure 1i). To investigate the potential role of FAM210B in ovarian cancer metastasis, we used orthotopic mouse models of ovarian cancer. Immunodeficient mice were injected with parental SKOV3-luc-GFP cells under the bursal membrane of the ovary, and peritoneal cavity metastases were developed after 4 weeks (Figure 1j). mRNA expression and immunohistochemistry (IHC) scores of FAM210B were higher in the metastatic site than in the primary ovarian cancer (Figures 1k and l). These findings showed the clinical significance and metastasis potential of FAM210B in cancer. To further study the role of FAM210B in human metastatic ovarian cancer, we assessed its expression in primary tumor sections from ovarian cancer patients and their matched metastatic tumors by IHC (Figure 2). Analysis of these tumor sections revealed diminished FAM210B staining (either signal intensity or percentage of positivity of FAM210B staining) in metastatic tumor sections compared with paired primary tumors in all eight patients examined.

### Inhibition of FAM210B Promoted Metastasis *in vitro* and *in vivo*

**Figure 3:**
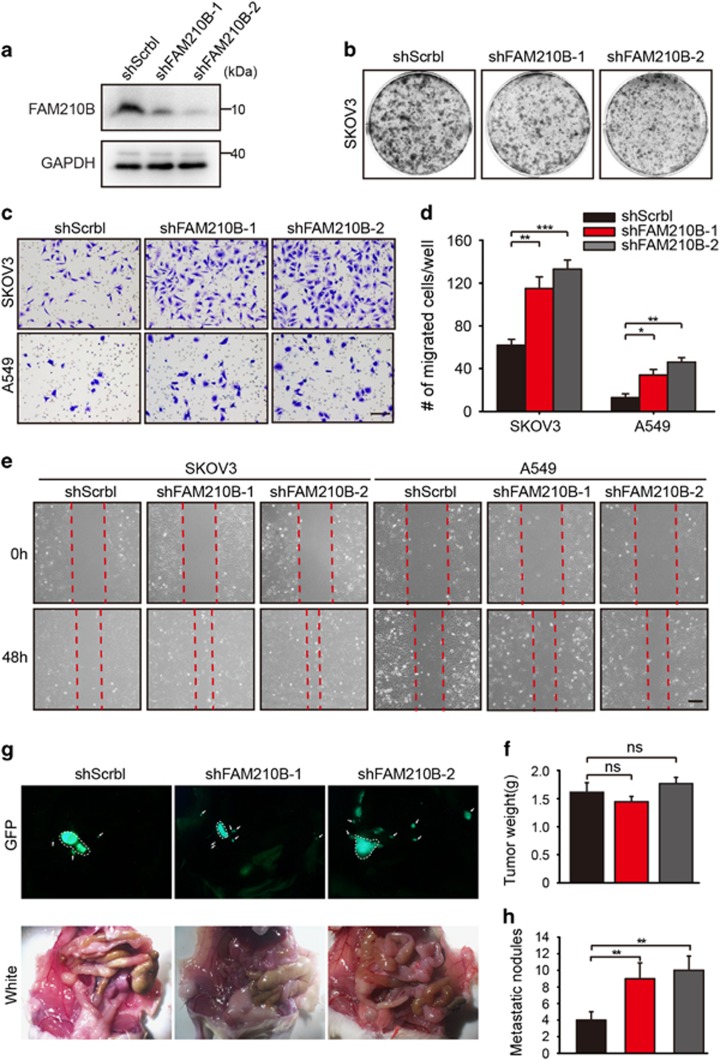
FAM210B expression modulates cancer cell invasion and migration. (**a**) Western blotting for FAM210B in the indicated cells/treatment. (**b**) Colony formation by the indicated cells. (**c**) Crystal violet-stained SKOV3 and A549 cells following invasion and (**d**) quantitation of invasion assay. (**e**) Light microscopic imaging (scale bar: 200 *μ*m) of migrated cells in the scratch assay. (**f**) SKOV3-luc-GFP and SKOV3-Luc-GFP-shFAM210B cells were injected under the bursal membrane of the ovary, and tumor weight was measured at the experimental end point (SKOV3-luc-GFP, *n*=6 mice; SKOV3-Luc-GFP-shFAM210B, *n*=6 mice, unpaired two-tailed Student’s *t*-test). (**g**) Representative images of mice harboring orthotopic SKOV3-luc-GFP and SKOV3-Luc-GFP-shFAM210B tumors. The primary tumors were indicated with white lines and the metastatic tumors were indicated with white arrows. (**h**) Number of peritoneal cavity metastasis nodules in mice with orthotopic SKOV3-luc-GFP and SKOV3-Luc-GFP-shFAM210B tumors. Data were represented as the means±S.E.M. NS: not significant. *P<0.05, **P<0.01, ***P<0.001, ****P<0.0001

Given that FAM210B loss was associated with metastasis and decreased survival, we used lentiviral shRNA to suppress FAM210B expression in SKOV3 and A549 cells and measured its effect on cell metastasis *in vitro* and *in vivo*. Figure 3a showed that the knockdown of FAM210B significantly decreased the protein levels in SKOV3. FAM210B knockdown significantly increased the invasion of cancer cells after 24 h (Figures 3c and d), whereas alterations in FAM210B expression had little effect on cell colony formation (Figure 3b and Supplemenetary Figure S1E). We also observed overexpression FMA210B in SKOV3 cells using lentivirus myc-FAM210B, and the invasion cells were less than control cells after 48 h (Supplemenetary Figures S3A and B). The migration assay was also dependent on the expression level of FAM210B (Figure 3e). SKOV3-Luc-GFP-shFAM210B orthotopic ovarian cancers showed similar tumor weight to control shScrbl tumors (Figure 3f). The number of pelvic cavity metastatic nodules was significantly increased in mice with SKOV3-Luc-GFP shFAM210B tumors compared with mice with control shScrbl tumors, as assessed by the number of GFP+ nodules (Figures 3g and h). Thus these results indicated that the loss of FAM210B increases the invasive and migratory properties of cancer cells.

### FAM210B was transported into and localized in mitochondria

**Figure 4:**
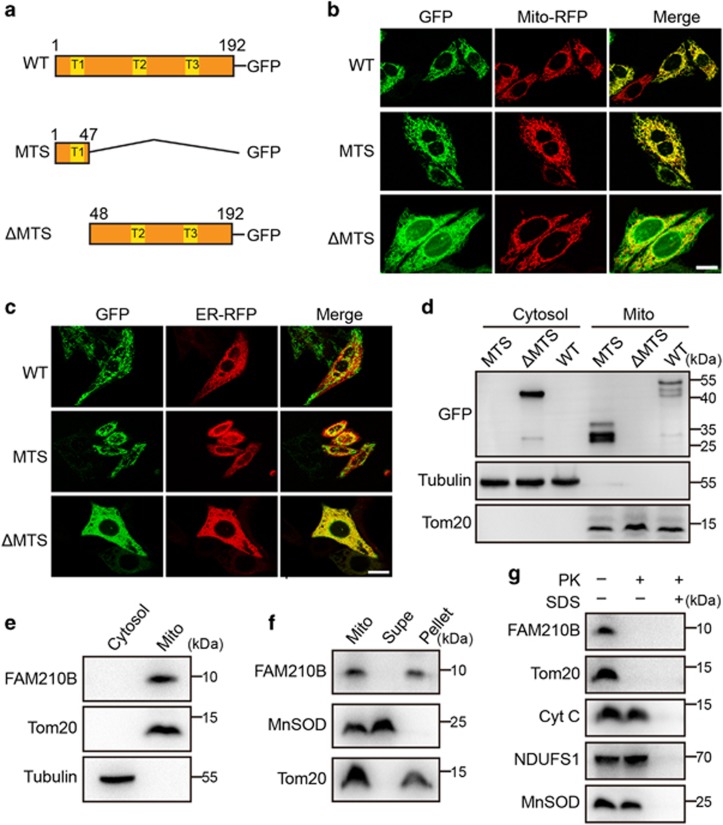
FAM210B is transported into and is localized in the mitochondria. (**a**) Schematic representation of FAM210B domains based on the primary structure of FAM210B. (**b**) Confocal microscopy of HeLa cells transfected with GFP-tagged human FAM210B and RFP-tagged mitochondria. FAM210B-GFP, GFP sequence introduced at the C terminus of FAM210B; FAM210B (MTS)-GFP, the MTS (aa 1–47) of FAM210B was added to the GFP N terminus; FAM210B (ΔMTS)-GFP, GFP was added to the C terminus of MTS (1–47)-deleted CRIF1. Scale bar, 20 mm. (**c**) Confocal microscopy of HeLa cells transfected with GFP-tagged human FAM210B and RFP-tagged endoplasmic reticulum. (**d**) Western blotting analysis following subcellular fractionation of GFP-tagged human FAM210B HeLa cells. (**e**) Western blotting analysis following subcellular fractionation of endogenous in HeLa cells. (**f**) The mitochondria of HeLa cells were swollen and sonicated to disrupt membranes, washed with alkali buffer (pH 11.5) to detach loosely associated proteins from membranes, and then re-isolated by ultracentrifugation. The supernatant (Supe) and membrane fractions (Pellet) were subjected to western blotting for FAM210B, TOM20, or MnSOD. (**g**) Mitochondria isolated from HeLa cells were subjected to proteinase K (PK) proteolysis to digest exposed proteins, and detergent (SDS) was used to disrupt both IMMs (inner membrane of mitochondria) and OMMs (outer membrane of mitochondria). The lysates were resolved and subjected to immunoblot analyses. The submitochondrial markers used are Tom20 (OMM), Cyt C (Cytochrome c, intermembrane space), NDUFS1 (IMM), and MnSOD (mitochondrial matrix)

FAM210B is a novel protein, and its cellular location and function are unclear. First, the protein expression of FAM210B was detected in various human cell lines (Supplemenetary Figure S3C). Based on its expression profile, we found that it was highly conserved among metazoa but not in yeast (Supplemenetary Figure S4A). Protein sequence analysis predicted a cleaved sequence located in the N-terminal region in human FAM210B that may serve as a mitochondrial targeting sequence (MTS) (Supplementary Figures S4B and C). According to the predicted analysis, C-terminal GFP-tagged FAM210B, MTS and ΔMTS constructs were constructed (Figure 4a). FAM210B-GFP and FAM210B (MTS)-GFP were exclusively localized in the mitochondria (Figure 4b), whereas FAM210B (ΔMTS)-GFP, an MTS-deleted FAM210B, was localized in the endoplasmic reticulum (Figure 4c) in HeLa cells, indicating that the 47 amino acid (aa) MTS of FAM210B was essential for protein mitochondrial targeting. To further confirm the mitochondrial localization of FAM210B, cellular cytosol and mitochondria were isolated and subjected to western blotting. FAM210B-GFP and FAM210B (MTS)-GFP were detected mostly in the mitochondrial fractions, whereas FAM210B (ΔMTS)-GFP was observed in the cytosolic fractions (Figure 4d). Immunoblotting of endogenous FAM210B expression also revealed that FAM210B was located in the mitochondria (Figure 4e). Furthermore, when mitochondria were sonicated for membrane disruption and then were washed, FAM210B was associated with disrupted mitochondrial pellets, suggesting that FAM210B was a mitochondrial membrane protein (Figure 4f). To further identify the submitochondrial localization, intact mitochondria isolated from HeLa cells were treated with proteinase K. FAM210B is sensitive to protease digestion similar to Tom20 and in contrast to NDUFS1 and MnSOD, which are resistant to degradation, indicating that endogenous FAM210B is localized on the outer membrane of mitochondria (Figure 4g). Taken together, these results showed that FAM210B resided on the outer membrane of mitochondria, and the N- terminal 47 aa MTS of FAM210B are necessary for its proper mitochondrial localization.

### Loss of FAM210B mediated metabolic reprogramming in cancer cells

**Figure 5:**
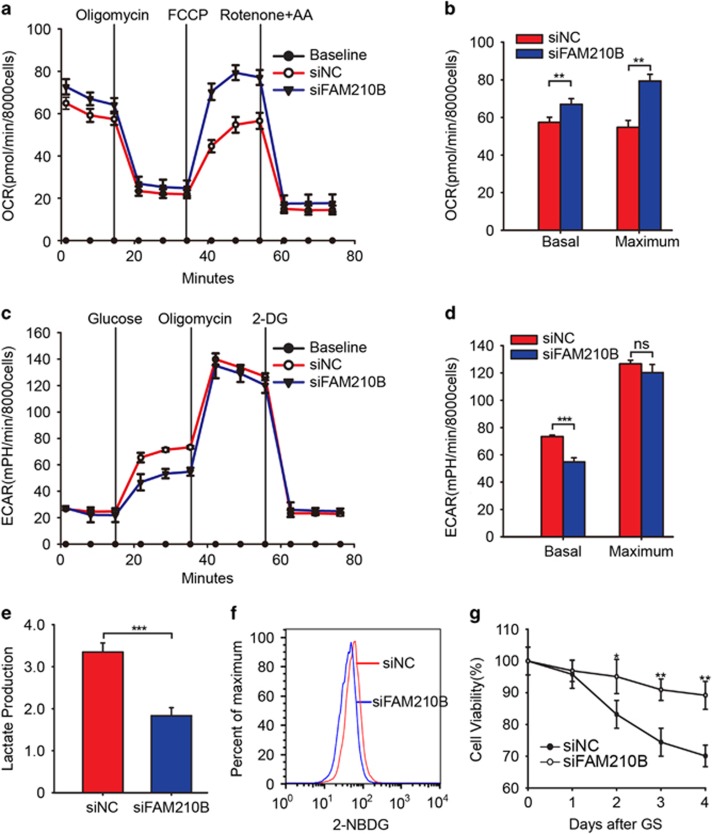
Loss of FAM210B mediates metabolic reprogramming in cancer cells. (**a**) Normalized OCR in siFAM210B (*n*=8) and negative control SKOV3 cells (*n*=8 wells). (**b**) Scale bar of basal OCR and maximum OCR in siFAM210B (*n*=8) and negative control SKOV3 cells (*n*=8 wells). (**c**) Relative ECARs normalized to the cell number over time in siFAM210B (*n*=8) and negative control SKOV3 cells (*n*=8 wells). (**d**) Scale bar of basal ECAR and maximum ECAR in siFAM210B (*n*=8) and negative control SKOV3 cells (*n*=8 wells). (**e**) Lactate production normalized to total cell protein in siFAM210B (*n*=3) and negative control SKOV3 cells (*n*=3). (**f**) Flow cytometry of 2-NBDG stain in siFAM210B and negative control SKOV3 cells. A total of 8000 cells was analyzed. (**g**) Cell viability rate normalized to day 1 in siFAM210B and negative control SKOV3 cells. Data were represented as the means±S.E.M. Statistics were compared using unpaired Student’s *t*-test; NS: not significant. *P<0.05, **P<0.01, ***P<0.001, ****P<0.0001

A recent study reported on the important roles of the reprogramming of cellular metabolism in tumor metastasis.^9^ Based on the mitochondrial localization of FAM210B and loss of the FAM210B effect on cancer metastasis, we measured its oxygen consumption rates (OCRs) and extracellular acidification rates (ECARs) in the presence or absence of distinct inhibitors. FAM210B was transiently silenced (gene expression knockdown; Stealth siFAM210B RNA pool) in SKOV3 and A549 cells. The baseline and maximum OCR were significantly increased in siFAM210B cells compared with the negative control in SKOV3 cells (Figures 5a and b) and A549 (Supplementary Figures S5A and B), while baseline ECAR levels were decreased, and the maximum ECAR levels were unchanged (Figures 5c, d, Supplemenetary Figures S5C and D). Consistently, siFAM210B cells showed reduced lactate production (Figure 5e) and higher glucose uptake (Figure 5f) and were also less sensitive to glucose deprivation (Figure 5g). However, total reactive oxygen species (ROS; Supplemenetary Figure S6A), mitochondria-derived ROS (Supplemenetary Figure S6B), mitochondrial respiratory activity (Supplemenetary Figure S6D), and mitochondrial structure (Supplemenetary Figure S6E) were unchanged in FAM210B-deficient cells while mitochondrial mass (Supplemenetary Figure S6C) was slightly increased. Thus siFAM210B cells exhibit metabolic reprogramming with higher Krebs cycle but with reduced baseline glycolysis compared with control cells.

### FAM210B knockdown decreased the expression of PDK4

**Figure 6:**
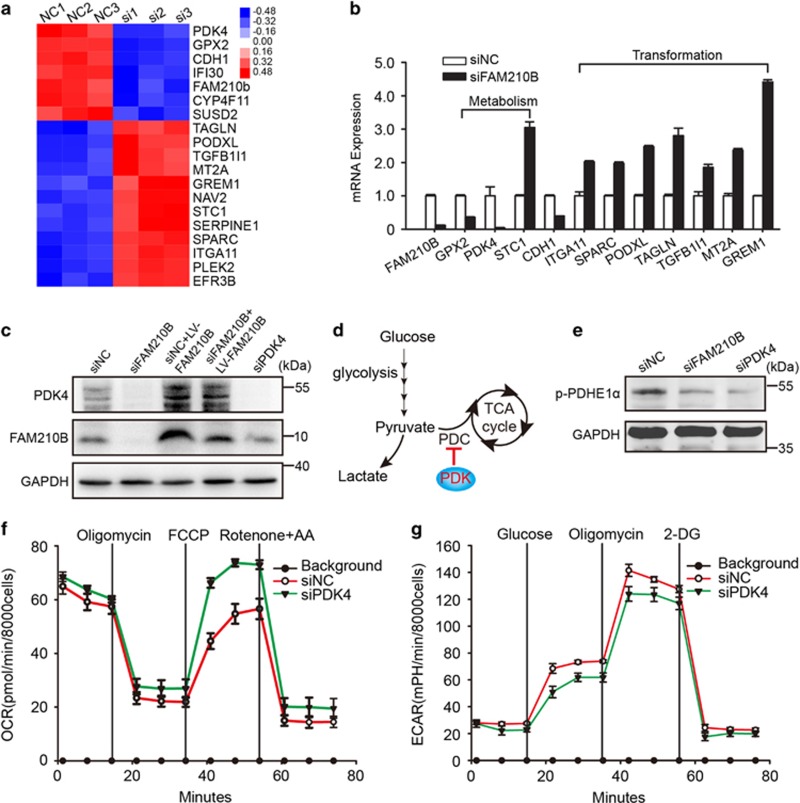
FAM210B knockdown cells show decreased PDK4 expression. (**a**) Heat map of the top 25 differentially regulated genes in siFAM210B and negative control A549 cells. (**b**) Quantitative PCR analyses of the relative expression of the indicated genes in siFAM210B normalized to negative control SKOV3 cells. (**c**) Immunoblotting for PDK4 in the indicated treated SKOV3 cells. (**d**) Schematically depicted PDK function in SKOV3 cell metabolism. (**e**) Western blotting analysis of the phosphorylation levels of PDH-E1*α* in the indicated treated SKOV3 cells. (**f**) Normalized OCR and (**g**) normalized ECAR in siPDK4 (*n*=6) and negative control SKOV3 cells (*n*=8 wells)

To further define the mechanism of FAM210B metabolic remodeling, the transcriptome of siFAM210B A549 cells and control cells were assessed by gene expression microarray. A heat map selected the top 25 differentially expressed genes in siFAM210B cells *versus* control, and PDK4 was dramatically downregulated (Figure 6a). Microarray data were verified by qRT-PCR in SKOV3 cells (*P*<0.001) (Figure 6b). Reduced expression of PDK4 in siFAM210B cells was partially restored via overexpressing FAM210B and was further validated by western blotting (Figure 6c). Nevertheless, we did not find a change in the expression of the other three pyruvate dehydrogenase (PDH) kinase genes (PDK1, PDK2, and PDK3) upon FAM210B knockdown (Supplemenetary Figure S7A). PDK4, a regulatory kinase, phosphorylates PDH to inhibit its activity (depicted schematically in Figure 6d). Therefore, the deficiency of PDK4 decreases glycolysis and activates mitochondrial energy metabolism.^13^ In line with this, the phosphorylation levels of PDH-E1*α* were attenuated after the reduced expression of FAM210B or PDK4 (Figure 6e). The suppression of PDK4 also showed a higher Krebs cycle but reduced baseline glycolysis compared with control cells (Figures 6f and g). Collectively, the data suggested that the metabolic and metastatic phenotype due to the knockdown of FAM210B may be PDK4 dependent.

### PDK4 decrease was essential for metabolic remodeling and metastasis due to FAM210B knockdown

**Figure 7:**
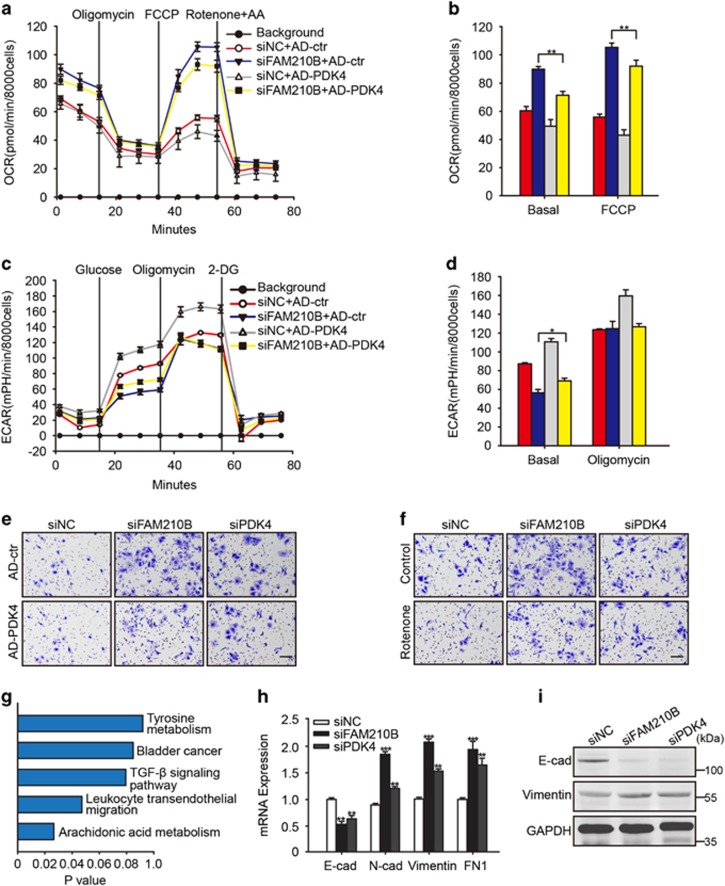
FAM210B knockdown was co-induced with an EMT program. Normalized OCR (**a**) and (**b**) scale bar of basal OCR and maximum OCR in the indicated cells (*n*=4). (**c**) Normalized ECAR and (**d**) scale bar of basal ECAR and maximum ECAR in the indicated cells (*n*=4). (**e** and **f**) Crystal violet-stained SKOV3 with different treatments. (**g**) KEGG pathway microarray analysis of different pathways in siFAM210B compared with the negative control. (**h**) mRNA expression of E-cadherin, N-cadherin, Vimentin, and FN1 in siFAM210B and siPDK4 cells *versus* control. (**i**) Protein expression of E-cadherin and Vimentin in the indicated cells. Data were represented as the means±S.E.M. NS: not significant. *P<0.05, **P<0.01, ***P<0.001, ****P<0.0001

Next, we wondered whether the loss of FAM210B-mediated metabolic features occurred through the decreased expression of PDK4. We overexpressed and validated PDK4 in siFAM210B cancer cells (Supplemenetary Figure S7B). As shown in Figures 7a and b, the basal and maximum OCR were partially reversed by restoring PDK4 gene expression in siFAM210B pooled cancer cells. Similarly, the basal ECAR was also partially restored (Figures 7c and d). To address the effects of the alteration of oxidative phosphorylation (OXPHOS) activity on the metastasis potential, we tested the roles of PDK4 knockdown or the mitochondrial complex I inhibitor, rotenone, on cancer cell invasion. Enhanced OXPHOS activity by siFAM210B or siPDK4 promoted invasion but was attenuated by PDK4 overexpression or rotenone (Figures 7e and f). To make clear the mechanism of cell invasion promoted by low expression of FAM210B, bioinformatics analysis of gene expression profiling revealed that TGF-*β* signaling differentially modulated the canonical pathway in siFAM210B compared with the control cells(Figure 7g). Mesenchymal genes (Vimentin and N-cadherin) were significantly upregulated while epithelial gene (E-cadherin) was downregulated in siFAM210B and siPDK4 cells compared with the control (Figures 7h and i). Together, these findings indicated that the downregulation of FAM210B assumed a characteristic EMT phenotype and remodeled metabolic features of mitochondrial OXPHOS.

## Discussion

Epithelial ovarian cancer (EOC) is the leading cause of death from gynecological malignancy, with a high mortality attributable to widespread intraperitoneal metastases.^14^ Therefore, we applied the suppression of mortality by SMART to screen genes involved in tumor progression. We showed that low expression of the new gene *FAM210B* attenuated the TSA effects in cancer cells and promoted malignant metastasis through metabolic reprogramming. More specifically, our findings demonstrated that (1) low expression of FAM210B significantly correlated with decreased survival and enhanced metastasis *in vivo* and *in vitro*; (2) FAM210B is a novel protein in the outer membrane of mitochondria; (3) loss of FAM210B cells displayed the metabolic signature of increased mitochondrial respiratory capacity and reduced glycolysis, due to the downregulation of PDK4; (4) PDK4 restoration or blockade of mitochondrial respiration in FAM210B knockdown cancer cells partially reversed their invasive potential; (5) FAM210B inhibition activated TGF-*β* signaling and subsequent EMT-associated gene expression.

Accumulating evidence has indicated that mitochondria had a central and multifunctional role in malignant tumor progression, and targeting mitochondria provides therapeutic opportunities.^15^ In our screen model, we demonstrated that the loss of a novel mitochondrial protein, FAM210B, was significantly associated with metastasis and decreased survival in a clinical setting. Furthermore, the reduced level of FAM210B was significantly correlated with decreased survival and enhanced metastasis *in vivo* and *in vitro* (Figure 3) in EOC, a finding that was not reported previously. FAM210B and its homologs belong to the domain of unknown function 1279 (DUF1279) family of proteins, and their functions in cells are poorly understood. A recent new study identified it as a putative mitochondrial protein using transiently transfected His/Myc-tagged FAM210B and to be involved in erythroid differentiation.^16^ Our observation revealed that different cell lines express FAM210B and FAM210B was an outer membrane protein of the mitochondria with an N-terminal 47 aa MTS (Figure 4). These findings suggest that the novel mitochondrial protein FAM210B may connect mitochondrial function to cancer progression.

A common feature of cancer cells is to utilize the nutrients to satisfy their needs. Thus targeting metabolic processes is attractive for therapy. Aerobic glycolysis is common in cancer cells and correlates with poor tumor prognosis.^17, 18^ However, glycolytic inhibitors have little effects on solid tumor growth.^19^ Another group assumed that altered metabolic and signaling cues in response to glycolysis inhibition in cancer cells promotes metabolic reprogramming and eventually the escape from glycolysis dependency.^20^ Recent studies have indicated that the migratory and invasive properties of cancer cells are dependent on mitochondrial respiration.^8, 9, 21^ Based on the mitochondrial location of FAM210B, we tried to explore whether its effect on metastasis is associated with mitochondrial apoptosis, biogenesis, respiratory chain activity, or mitochondrial ROS; however, the results are negative (Supplemenetary Figure S6). Next, we wondered whether mitochondrial metabolism contributes to the metastasis feature in FAM210B knockdown cells. We measured the OCR and ECAR in the presence or absence of distinct inhibitors and found that the loss of FAM210B cells showed the metabolic signature of increased mitochondrial basal OCR and respiratory capacity but reduced glycolysis (Figure 5). The phenomenon is in agreement with recent work reflecting the metabolic plasticity of tumor cells.^20, 22^ Knockdown of FAM210B cancer cells with high OXPHOS but relatively low basal ECAR showed some differences from the others. Microarray revealed that the reduced expression of FAM210B in cancer cells also showed a lower expression of PDK4 (Figure 6). This may explain the negative results of mitochondrial apoptosis, biogenesis, and respiratory chain activity and only effects on OCAR in FAM210B knockdown cells. However, the contribution of the mechanism of metabolism reprogramming to metastasis is not thoroughly clarified.

Settleman and colleagues described that PDK4 downregulation changes pyruvate entry into the TCA cycle and is a critical regulator of EMT.^23^ KEGG pathway analysis of the microarray data demonstrated that the loss of FAM210B activates TGF-*β* signaling and subsequent EMT-associated gene expression, and we also confirmed the phenomenon in FAM210B or PDK4 knockdown cells (Figures 7g–i). Focal hypoxia and ROS accumulation may induce an EMT program.^24, 25^ However, the decreased expression of FAM210B or PDK4 demonstrated little to no effect on mitochondrial-derived ROS. On the other hand, PDK4 restoration or blockade of mitochondrial respiration in FAM210B knockdown cancer cells partially reversed their invasive potential (Figures 7a–f). Collectively, we propose that the EMT program and enhanced migratory and invasive properties may coexist with mitochondrial reprogramming in FAM210B or PDK4 knockdown cancer cells, while the mechanism needs further clarification.

In this study, we successfully exploited the SMART technique to identify a novel cancer progression-suppressor gene, *FAM210B*, and demonstrated that it encodes an outer mitochondrial membrane protein and is related to cancer metabolism reprogramming and metastasis. Our results offer a mechanistic rationale for the further investigation of FAM210B as a potential therapy to prevent or treat cancer metastasis.

## Materials and Methods

### Cell culture and transfection

HeLa (human cervical adenocarcinoma), A549 (human lung carcinoma), and SKOV3 (human ovarian adenocarcinoma) cell lines were obtained from ATCC, USA. HeLa and A549 cells were cultured in DMEM (Invitrogen), and SKOV3 were maintained in McCoy’s 5a (Invitrogen) supplemented with 10% FBS (Gibco, Grand Island, NY, USA) and 100 U/ml penicillin and streptomycin (Invitrogen) at 37 °C in a humidified incubator with 5% CO_2_. For the stable RNA interference of FAM210b and PDK4, predesigned shRNAs from Shanghai Genechem of China were used, and puromycin-resistant clones were subsequently propagated. Specific Stealth siRNA pool targeting Fam210B and Pdk4 were custom designed and provided by Invitrogen. Stealth siRNA pool transfection used Lipofectamine 3000 (Invitrogen) according to the manufacturer’s instructions. The Stealth siRNA sequences were as follows: FAM210B: 5′-AAUAUCUGACAAUCAAGGGCACAGA-3′, 5′-UAGGCCACCACGAAGGUACUUGUGC-3′, 5′-UCUCACUGGCGCAAACAGCUUGUGG-3′ PDK4: 5′-UUAAGUAGAUGAUAGCAUCUGUUCC-3′, 5′-AAGAGGUAUUUACUAAUUGGGUCGG-3′, 5′-AAAUCCAUCAGGCUCUGUAUAUACC-3′. For the overexpression of PDK4, recombinant adenovirus expressing PDK4 and empty control adenovirus were constructed by Shandong Vigene Biosciences. For the overexpression of FAM210B, recombinant lentivirus expressing myc-FAM210B and myc-control lentivirus were also from Shandong Vigene Biosciences of China

### Plasmids and expression constructs

Full-length FAM210b, N-terminal 47 aa and the 48–192 aa of FAM210b constructs were developed using human FAM210B complementary DNA clone (OriGene, Rockville, USA, SC124466) and PCR, and the PCR products were cloned into pGFP-N1 vector (Clontech, USA). Mitochondrial or endoplasmic reticulum morphology and fusion were observed using Mito-DsRed (Clontech) or ER-DsRed (Clontech).

### Mitochondrial isolation, extraction of mitochondrial proteins, proteolysis of mitochondria fraction and western blotting analysis

Mitochondria isolation was performed using a mitochondria isolation kit (Thermo Scientific, Rockford, IL, USA) according to the manufacturer’s instructions. The extraction of mitochondrial proteins was performed as previously described.^26^ The proteolysis of mitochondria was performed as previously described with some modifications.^27^ Briefly, purified mitochondrial pellets were treated as follows: proteinase K (0.1 mg/ml), 1% SDS (membrane solubilization) at 37 °C and centrifuged at 12 000 × *g* for precipitation. Western blotting analysis was determined according to standard protocols with commercially available antibodies: anti-GFP (Abcam, ab6556), anti-*α*-Tubulin (Proteintech Group, Wuhan, Hubei, P.R.C, 11224-1-AP), anti-Tom20 (Proteintech Group, Wuhan, Hubei, P.R.C, 11802-1-AP), anti-Fam210b (Abcam, Cambridge, UK, ab150889 and Santa Cruz, CA, USA, sc-85318), anti-SOD2 (R&D Systems, MN, USA, MAB3419), anti-Cytochrome *c* (R&D Systems, MN, USA, MAB897), anti-NDUFS1 (Proteintech Group, 12444-1-AP), anti-GAPDH (Proteintech Group, 60004-1-Ig), and anti-PDK4 (Abcam, ab110336).

### Microarray data analysis and mining

Cells were homogenized with TRIzol (Invitrogen) and submitted to Genminix Informatic Ltd. (Shanghai, China). Microarray analysis was performed using Affymetrix Human Transcriptome Array 2.0 (HTA2.0, Affymetrix, CA, USA). The data were analyzed with Robust Multichip Analysis algorithm using Affymetrix default analysis settings and global scaling as normalization method. Gene expression validation by real-time PCR was performed using the primers listed in Supplementary Table S1. The heat map was drawn using Cluster 3.0. Gene expression data and survival analyses of ovarian, breast, and lung cancer patients with annotated clinical outcomes were downloaded from the Gene Expression Omnibus database (GSE9187, GSE37344, GSE14407, GSE19829, GSE9891, and GSE11969). For survival analyses, the median gene expression value was used to define the high and low Fam210b expression groups, and KM analyses were performed. Significance for these plots was evaluated using the log-rank test.

### *In vivo* animal studies

For the orthotopic mouse model of ovarian cancer studies, 4-week-old female NOD-SCID mice were purchased from and were housed at the Experimental Animal Center, Tongji Medical College, HUST, Wuhan, China. The orthotopic mouse model of ovarian cancer was performed as previously described.^28^ For the development of the pelvic cavity metastasis study, mice were surveyed by *in vivo* imaging for the tumor growth curve or by stereomicroscopy for GFP+ nodules in tumor implantation after 1–4 weeks.

### Invasion and migration assays

For the invasion assay, the polycarbonate membranes (transwell; 8-*μ*m pore) were coated on both sides with Matrigel (BD Biosciences, USA), and the cells were seeded in the upper chamber, and the migrated cells on the basal side of the membrane were fixed in 4% paraformaldehyde and were stained with crystal violet before microscopic evaluation. For the scratch/migration assay, the cell-free area was measured 24 h after scratching the dish, and 0.1 *μ*M rotenone was used.

### Clonogenic assay and MTT

After treatments, 2 × 10^3^ cells were plated in 6-cm culture dishes for 14–20 days. Colonies were fixed with 4% paraformaldehyde, stained with 2% crystal violet, and counted. For the cell viability MTT assay, treated cells were seeded in 96-well plate for 24–72 h with or without the indicated concentrations of TSA (Sigma, St. Louis, MO, USA). The MTT assay was performed as described previously.^29^

### Measurement of the OCR and ECAR

The OCR and ECAR were measured using the Seahorse XF24 and XF96 instruments (Seahorse Bioscience, USA). Next, 2.5 × 10^4^ cells were plated in XF24 Cell Culture Microplates and 8000 cells in XF96. For the OCR test, the cell medium was changed by the assay medium (Seahorse Bioscience) supplemented with 2 mM glutamine, 10 mM glucose, and 1 mM pyruvate for 1 h in a 37 °C incubator prior to the measurements using the XF Cell Mito Stress Kit (Seahorse Bioscience). The concentrations of oligomycin and FCCP were 1.0 and 0.5 *μ*M FCCP for SKOV3 and A549 cells, respectively. For the glycolytic metabolism test, the cells were incubated in the basal medium (Seahorse Bioscience) for 1 h in a 37 °C incubator prior to measurement using the Glycolytic Stress Test Kit (Seahorse Bioscience). The OCR and ECAR results were adjusted to the cell numbers. Finally, the cells that were prepared but not undergone any treatment were stained with CCK8 following spectrophotometric measurements at 450 nm (Molecular Device, USA).

### Analysis of lactate production

Cell culture supernatants were tested following the manufacturer’s instructions (Lactate Colorimetric/Fluorometric Assay Kit, BioVision, CA, USA), and the data were normalized to the total protein.

### Mitochondrial DNA (mtDNA) content determination

mtDNA content measurement was performed as previously described.^30^ Briefly, the relative mtDNA content was measured by quantitative real-time PCR analysis of total DNA extracted from cells by assessing the relative levels of the *MT-ND1* gene in mtDNA (F: 5′-CCCTAAAACCCGCCACATCT-3′ and R: 5′-GAGCGATGGTGAGAGCTAAGGT-3′) *versus* nuclear gene human globulin (F: 5′-GTGCACCTGACTCCTGAGGAGA-3′ and R: 5′-CCTTGATACCAACCTGCCCAG-3′).

### Measurement of mitochondrial superoxide, glucose uptake and mitochondrial respiratory chain enzyme activity

For mitochondrial superoxide measurement, the cells were incubated with 5 *μ*M MitoSOX (Molecular Probes) for 15 min at 37 °C and were measured by flow cytometry at FL2. For glucose uptake, the cells were incubated with 20 *μ*M 2-NBDG (Molecular probes, Thermo Scientific, USA) for 30 min at 37 °C and were measured by flow cytometry at FL1. Mitochondrial respiratory chain enzyme activity was detected using the Complex I, II and IV Enzyme Activity Microplate Assay Kit (MitoSciences, OR, USA, MS141, MS241, MS441) and ATP synthase Enzyme Activity Microplate Assay Kit (MitoSciences, MS541) according to the manufacturer’s instructions.

### Transmission electron microscopy

The cells were collected and fixed with 2.5% glutaraldehyde in 0.1 mol/l sodium cacodylate buffer and then were postfixed with 2% osmium tetroxide in 0.1 mol/l sodium cacodylate buffer plus 0.3% potassium ferrocyanide. The cells were stained with 4% aqueous uranyl acetate, dehydrated, infiltrated, and embedded in epoxyresin. Ultrathin sections (80 nm) were cut and imaged with an aFEI Tecnai G^2^ electron microscope (Tongji Hospital, Wuhan, PRC).

### Immunohistochemistry

Tissues were subjected to standard heat-induced epitope retrieval in 10 mM citrate buffer (pH 6.0) using a steamer. The slides were incubated overnight at 4 °C with a rabbit anti-Fam210b (Abcam, ab150889). Following incubation with the primary antibody, the secondary biotin-conjugated antibody was applied for 1 h at 37 °C. All of the slides were counterstained using Harris hematoxylin (Abcam).

### Statistical analysis

The statistical significance of the treatment groups was determined using the independent samples *t*-test or one-way ANOVA. All of the analyses were conducted using SPSS19.0 (USA).
