# Correction: Loss of the novel mitochondrial protein FAM210B promotes metastasis via PDK4-dependent metabolic reprogramming

**DOI:** 10.1038/s41419-019-1945-y

**Published:** 2019-09-23

**Authors:** Shujuan Sun, Jia Liu, Meisong Zhao, Yingyan Han, Pingbo Chen, Qingqing Mo, Beibei Wang, Gang Chen, Yong Fang, Yuan Tian, Jianfeng Zhou, Ding Ma, Qinglei Gao, Peng Wu

**Affiliations:** 1 The Key Laboratory of Cancer Invasion and Metastasis of the Ministry of Education of China, Tongji Hospital, Tongji Medical College, Huazhong University of Science and Technology, Wuhan, Hubei 430030 P. R. China; 2 Department of hematology, Tongji Hospital, Tongji Medical College, Huazhong University of Science and Technology, Wuhan, Hubei P. R. China

**Correction to: Cell Death & Disease**

10.1038/cddis.2017.273, published online 8 June 2017

Following publication of this article [1], the authors became aware of an error in Fig. 7e which requires correction. The images do not currently match the correct treatment and/or control conditions. Specifically, the images of siNC+AD-ctr (the top left panel) and siPDK4+AD-PDK4 (the bottom right panel) were incorrect. The corrected figure is provided below. The error does not impact the conclusions of the article. They sincerely apologize for the mistakes in the article and any inconvenience caused.

**Fig. 7:**
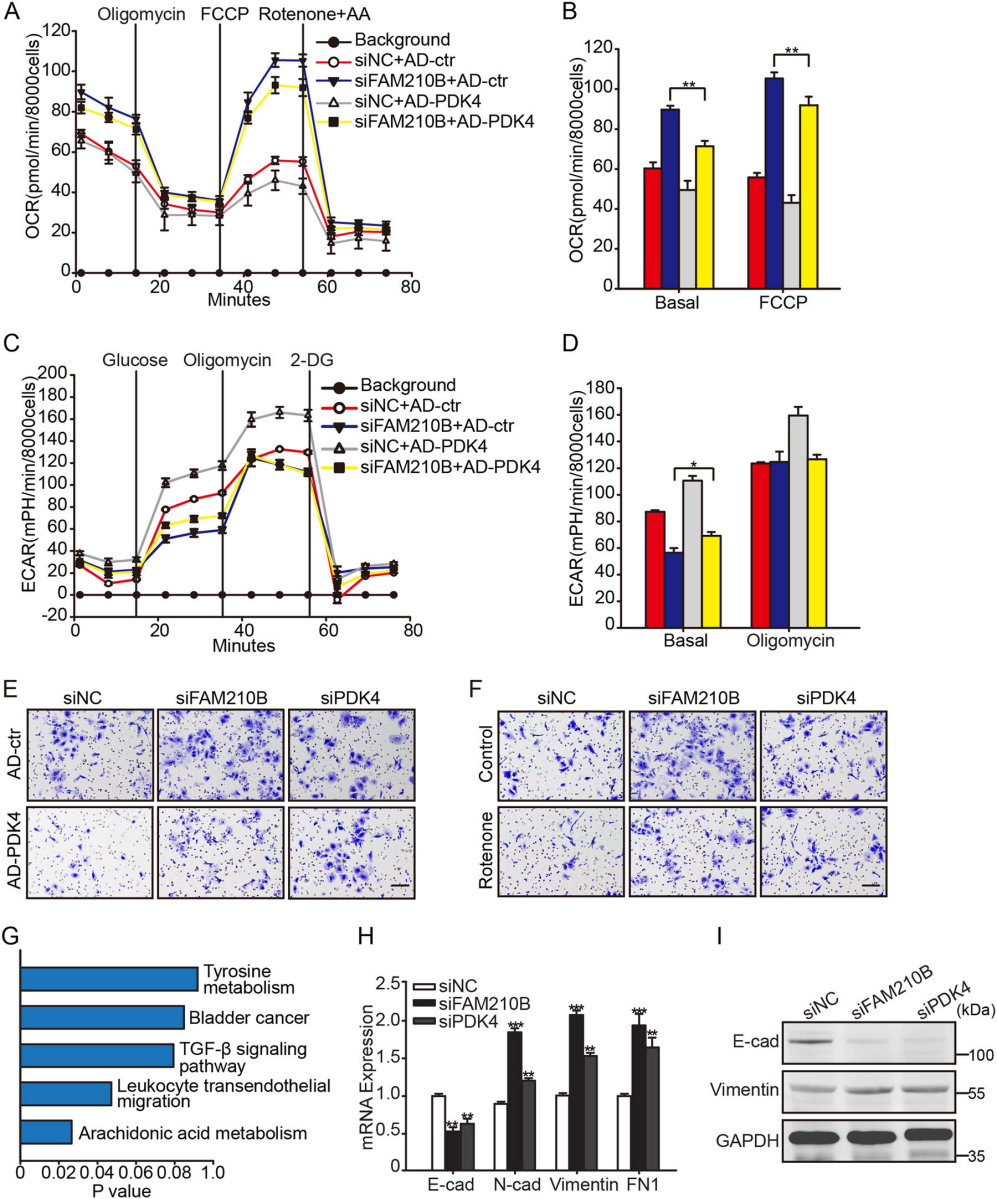
FAM210B knockdown was co-induced with an EMT program. Normalized OCR (**a**) and (**b**) scale bar of basal OCR and maximum OCR in the indicated cells (*n* = 4). **c** Normalized ECAR and (**d**) scale bar of basal ECAR and maximum ECAR in the indicated cells (*n* = 4). **e**, **f** Crystal violet-stained SKOV3 with different treatments. **g** KEGG pathway microarray analysis of different pathways in siFAM210B compared with the negative control. **h** mRNA expression of E-cadherin, N-cadherin, Vimentin, and FN1 in siFAM210B and siPDK4 cells *versus* control. **i** Protein expression of E-cadherin and Vimentin in the indicated cells. Data were represented as the means ± S.E.M. NS not significant. *P < 0.05, **P < 0.01, ***P < 0.001, ****P < 0.0001

