# Supplementary material for: Loss of the novel mitochondrial protein FAM210B promotes metastasis via PDK4-dependent metabolic reprogramming
Source: Cell Death Dis. 2017 Jun 8;8(6):e2870–. doi: 10.1038/cddis.2017.273 (PMC5520928; doi:10.1038/cddis.2017.273)
Supplement: Supplementary Information [file cddis2017273x1.docx]

**Supplementary information**

**
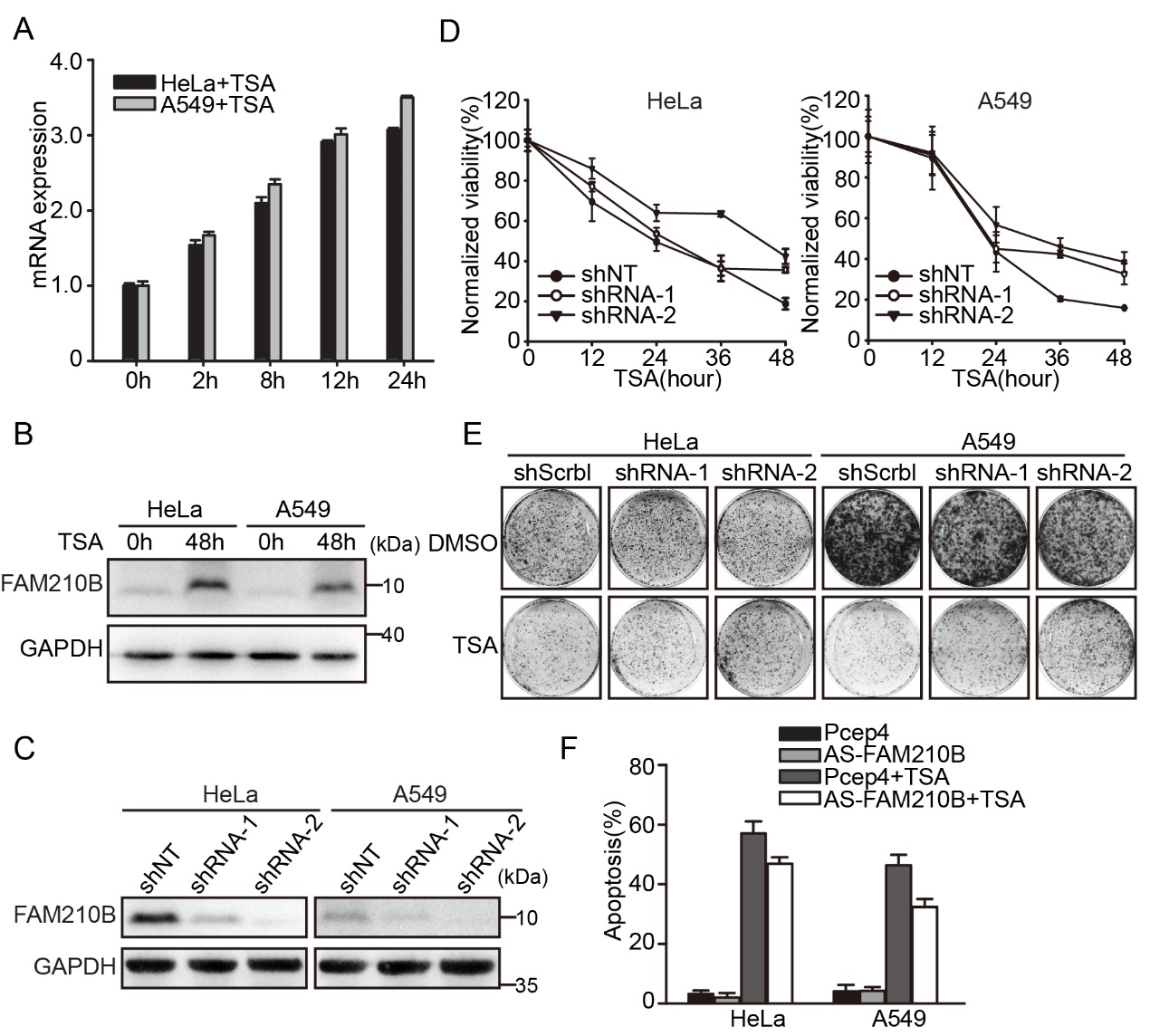
**

**FigureS1. TSA increases the transcription of FAM210B, and the level of FAM210B modulates the apoptosis induced by TSA.** (A) Levels of FAM210B mRNA assessed in HeLa or A549 cells treated with TSA at the indicated time using qRT-PCR (n = 3). **(B)** Immunoblotting of FAM210B in HeLa or A549 cells treated with TSA at the indicated time. **(C)** HeLa or A549 cells were transfected with the indicated constructs and were subjected to Western blot analysis after 72 h. (D) Cell viability of HeLa or A549 cells transfected with the indicated constructs with 250 nM TSA at the indicated time using MTT(n=4). (E) Cell colony formation of HeLa or A549 cells transfected with the indicated constructs with 250 nM TSA. (F) Apoptosis of HeLa or A549 cells transfected with the indicated constructs after treated with TSA.

**
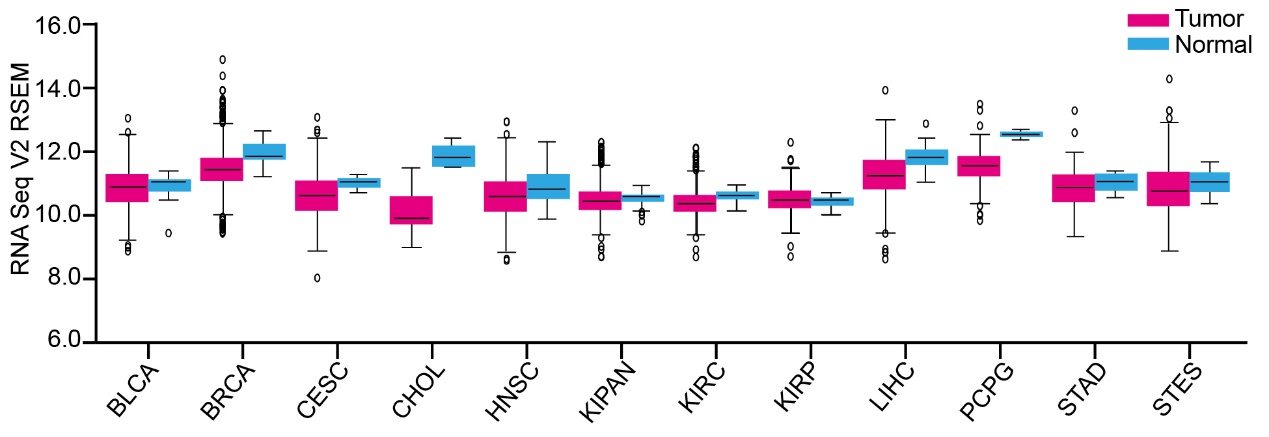
**

**Figure S2. The mRNA level of FAM210B in different normal and pared tumor tissues in TCGA data.**

**
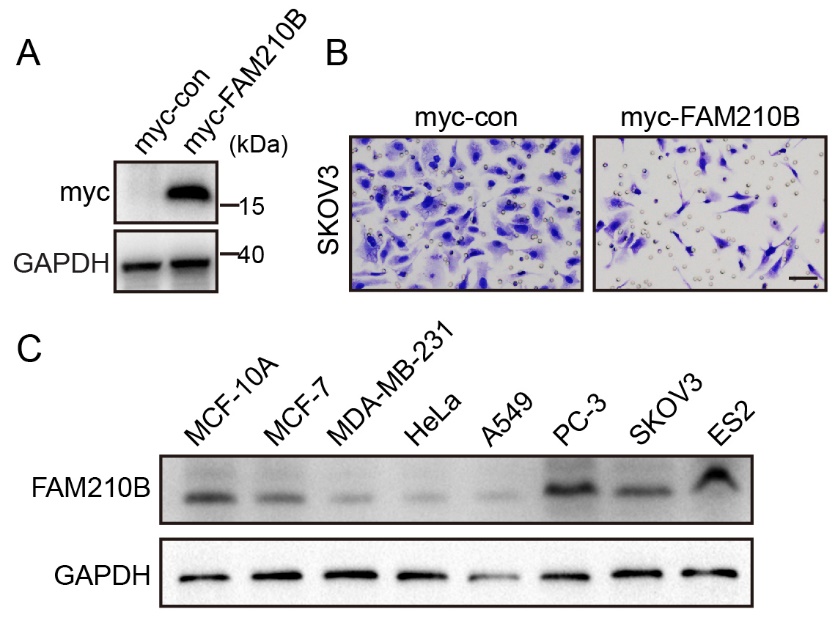
**

**Figure S3. Overexpression of FAM210B effects on SKOV3 invasion and protein Level of endogenous FAM210B in Different Cell Lines.** **(A)** Immunoblotting of myc in SKOV3 cells transfected with lentivirus expressing myc-FAM210B and myc-control lentivirus after 48 hours. **(B)** Crystal violet-stained SKOV3 cells after 48 hours under indicated treatments. (scale bar: 50μm). **(C)** Level of endogenous FAM210B in different cell lines.

**
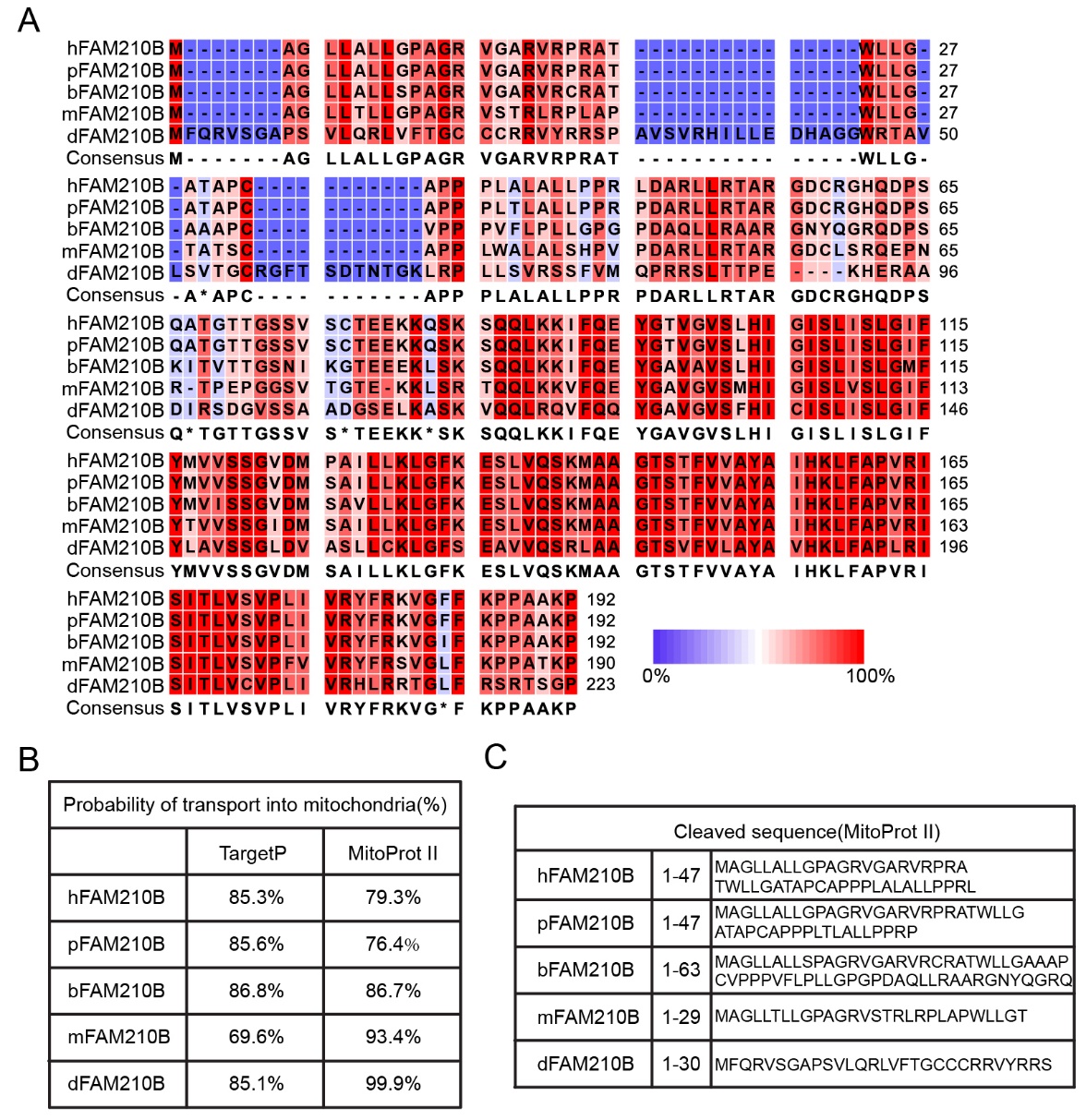
**

**Figure S4. Protein analysis of FAM210B homologs in different species.** hFAM210B, human FAM210B; pFAM210B, pan troglodytes FAM210B, bFAM210B, bovine FAM210B; mFAM210B, mouse FAM210B; dFAM210B, drosophila FAM210B; (A) Multiple amino acid sequence alignment in FAM210B proteins (CLC Sequence Viewer 5). (B) Probability of FAM210B transport into mitochondria. (C) Predicted mitochondrial targeting sequences in the N-terminus of FAM210B.


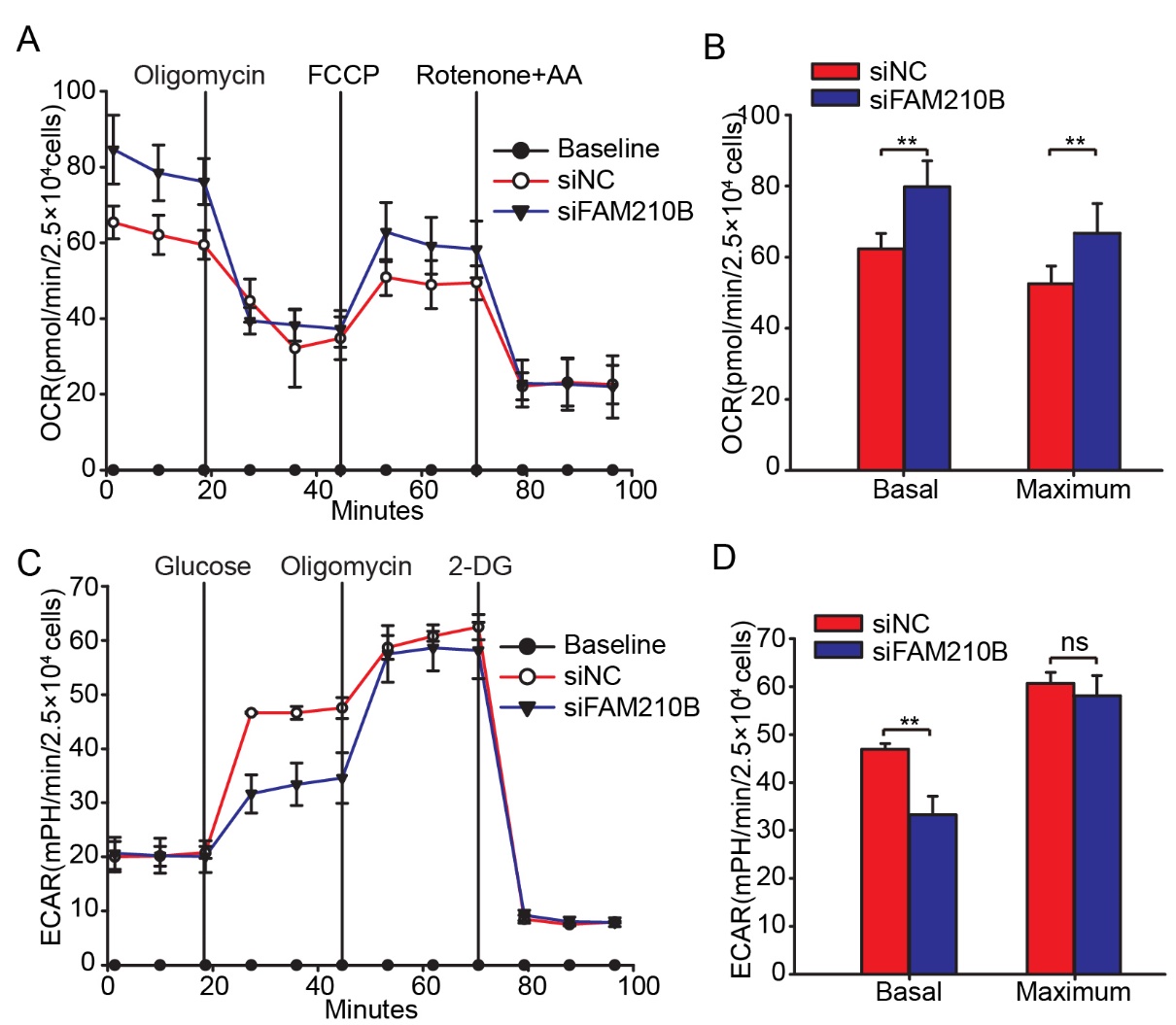


**Figure S5. Loss of FAM210B mediates metabolic reprogramming in A549.** (A) Normalized oxygen consumption rate (OCR) in siFAM210B (n=8) and negative control A549 cells (n=4 wells). (B) Scale bar of basal OCR and maximum OCR in siFAM210B (n=8 wells) and negative control A549 cells (n=4 wells). (C) Relative extracellular acidification rates (ECARs) normalized to the cell number over time in siFAM210B (n=4 wells) and negative control A549 cells (n=4 wells). (D) Scale bar of basal ECAR and maximum ECAR in siFAM210B (n=4 wells) and negative control A549 cells (n=4 wells).


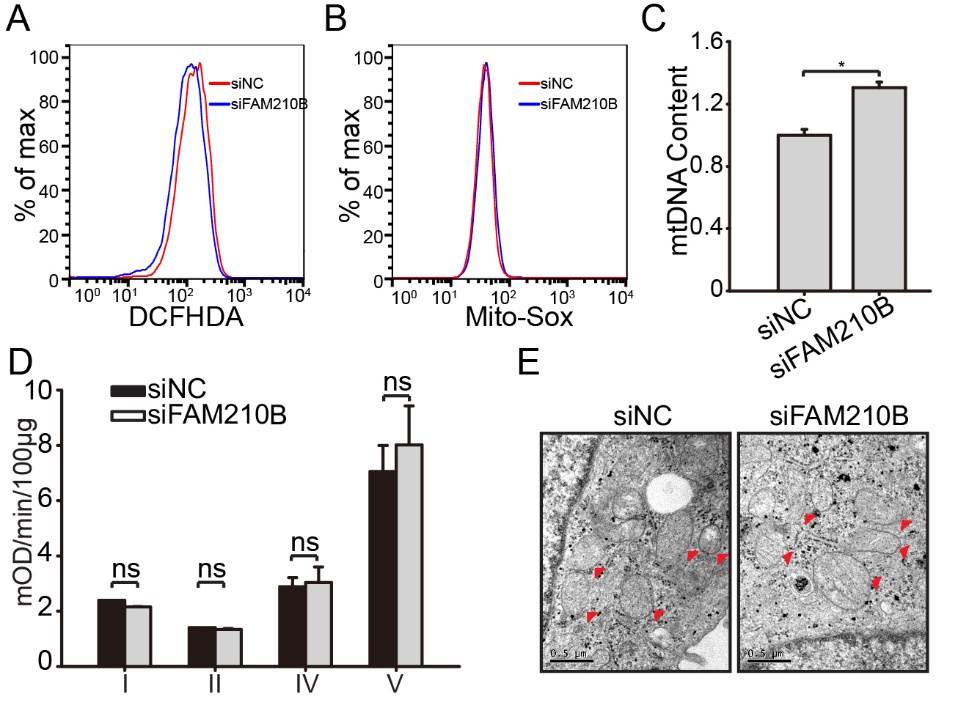


**Figure S6. Mitochondrial ROS, mitochondrial mass, mitochondrial complex activity and mitochondria structure in FAM210B knockdown Cells.** (A) Fluorescence of DCFHDA as a measure of total ROS levels in cells. n = 3. (B) Fluorescence of MitoSox as a measure of mitochondrial ROS levels. n = 3. (C) qPCR analysis of total DNA extracted from cells by assessing the relative levels of the MT-ND1 gene in mtDNA. (D) Measurement of the activities of mitochondrial OXPHOS complexes. n=3. (E) Transmission electron microscopy shows the appearance of mitochondria in the indicated treatments. Scale bar, 500 nm.


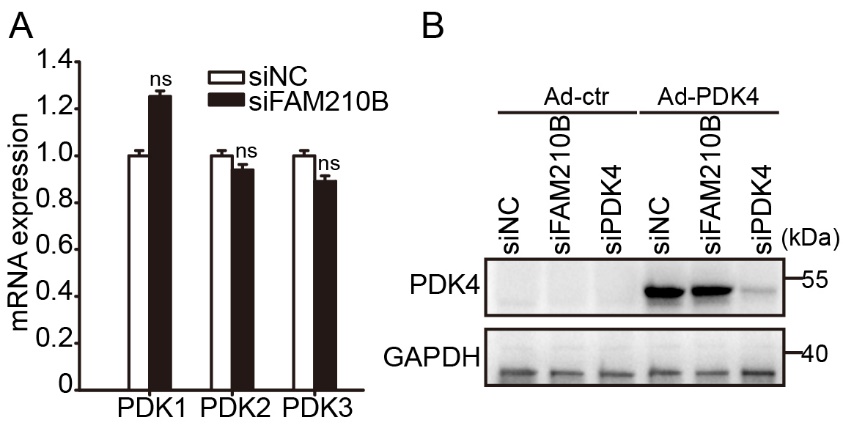


**Figure S7. PDK expression in FAM210B knockdown cells.** (A) qPCR analysis of PDK1, PDK2 and PDK3 in the indicated cells. (B) Protein level of PDK4 in different treatment cells.

**Supplementary Table 1. Summary of real-time PCR primer sequences for human genes.**

| Gene | Forward primer(5'-3') | Reverse primer(5'-3') |
| --- | --- | --- |
| *FAM210B* | CCGGAATTCATGGCCGGGTTGCT | CCGCTCGAGAGGTTTTGCAGCTG |
| *GPX2* | CCCTTGCAACCAATTTGGAC | TCCTTCAGGTAGGCGAAGAC |
| *PDK4* | AATGCTCCTTTGGCTGGTTT | GGCTGACTTGTTAAAAACTGGAA |
| *PDK3* | GCCAATTTCCCGTCTGTATG | CGCCATGCGGACTTATTAAA |
| *CDH1* | CACCCTGGCTTTGACGCCGA | AAAATTCACTCTGCCCAGGACGCG |
| *TGFB1I1* | TACAGCACGGTATGCAAGCC | GCAACCGATCTAGCTCACAGAG |
| *PDK1* | GCAGCAGAGAGTAAACTGTTTG | TGGTCACCTGACCTCTCG |
| *PDK2* | TGGACCGCTTCTACCTCAG | TCTTTCACCACATCAGACACG |
| *STC1* | CACACCCACGAGCTGACTTC | TCTCCCTGGTTATGCACTCTCA |
| *GREM1* | AAGCGAGACTGGTGCAAAAC | CTTGCAGAAGGAGCAGGACT |
| *CDH2* | CGCCATCCAGACCGACCCAA | GTCGATTGGTTTGACCACGGTGAC |
| *FN1* | CGGTGGCTGTCAGTCAAAG | AAACCTCGGCTTCCTCCATAA |
| *VIM* | GACGCCATCAACACCGAGTT | CTTTGTCGTTGGTTAGCTGGT |
| *TAGLN* | GAGCAAGCTGGTGAACAGCC | GACCATGGAGGGTGGGTTCT |
| *GAPDH* | GGACCTGACCTGCCGTCTAG | GTAGCCCAGGATGCCCTTGA |
